# Genome Analysis for Sequence Variants in SARS-CoV-2 Among Asymptomatic Individuals in a Long-term Care Facility

**DOI:** 10.1001/jamanetworkopen.2021.7939

**Published:** 2021-04-09

**Authors:** Baha Abdalhamid, Peter C. Iwen, Michael R. Wiley, Catherine B. Pratt, Steven H. Hinrichs

**Affiliations:** 1 Department of Pathology and Microbiology, University of Nebraska Medical Center, Omaha; 2 Nebraska Public Health Laboratory, University of Nebraska Medical Center, Omaha; 3 Department of Environmental, Agricultural and Occupational Health, College of Public Health, University of Nebraska Medical Center (UNMC), Omaha,

## Abstract

This cohort study analyzes sequence variants in the genome of SARS-CoV-2 using viral RNA samples taken from 7 women at a long-term care facility.

## Introduction

Studies have suggested that sequence variants in the genome of SARS-CoV-2 may affect infectivity, transmission, and pathogenicity of the virus.^1,2,3^ In this study, genome analysis was performed on SARS-CoV-2 RNA recovered from 7 individuals in a long-term care facility who were asymptomatic at time of screening.

## Methods

This cohort study followed the Strengthening the Reporting of Observational Studies in Epidemiology (STROBE) reporting guideline. As per 45 CFR 46.102(I), this study did not require University of Nebraska Medical Center institutional review board approval because it supported the COVID-19 public health response. No informed patient consent was required because the individuals were deidentified and institutional review board approval was not required.

After contact with a health care worker positive for SARS-CoV-2, 20 female residents in a long-term care facility were screened for SARS-CoV-2 symptoms and a nasopharyngeal swab was collected. At the time of screening, all the residents were asymptomatic with 7 subsequently identified as positive for SARS-CoV-2 by a commercial polymerase chain reaction (PCR) assay. A second specimen was collected from all 7 individuals, deidentified, and retested using the US Centers for Disease Control and Prevention nCOV PCR assay. Whole genome sequencing (WGS) was performed using the MinION platform.^4^ After uploading consensus genomes to the GISAID database, Nextclade version 0.9.0 was used for quality checks and data analysis (Table). Data analysis was performed from September to December 2020.

**Table.** Characteristics of SARS-CoV-2 Strains Detected in Asymptomatic Individuals From Long-term Care Facility

Sample ID^a^	N1, Ct^b^	N2, Ct^b^	GISAID ID^c^	NextClade^d^
NE-001-17	22.42	23.2	EPI_ISL_732820	20C
NE-002-17	26.61	27.31	EPI_ISL_732821	20C
NE-003-17	26.55	27.27	EPI_ISL_732822	20C
NE-004-17	18.94	19.58	EPI_ISL_732823	20C
NE-005-17	17.09	17.66	EPI_ISL_732824	20C
NE-006-17	22.91	23.49	EPI_ISL_732825	20C
NE-007-17	17.2	17.72	EPI_ISL_732826	20C

Abbreviation: Ct, cycle threshold.

^a^ All 7 asymptomatic individuals were female. Their samples were collected on September 26, 2020.

^b^ N1 and N2: Regions of SARS-COV-2 nucleocapsid (N) genes detected by polymerase chain reaction.

^c^ Accession ID of SARS-CoV-2 strains uploaded to GISAID database.

^d^ All 7 SARS-CoV-2 strains were grouped in 20C by NextClade.

## Results

The 7 asymptomatic female individuals with PCR positive results, whose ages ranged from 75 to 92 years, had PCR cycle threshold values that ranged from 17 to 27 (Table), reflecting high viral loads. During the 30 days following confirmation of COVID-19 infection, 5 individuals remained asymptomatic while 2 individuals developed lethargy and cognitive decline with no respiratory symptoms and both died after 2- and 4-weeks, respectively. The study group had less than 3 nucleotide substitutions among strains but 13 to 15 substitutions when compared with Wuhan-Hu-1 reference strain (GenBank NC045512), grouping them into clade 20C. The S:D614G variant within the spike (S) protein was present in all 7 genomes. The other variants resulted in 7 or 8 amino acid (AA) differences at the protein level (Figure). Two of the nonconserved amino acid changes were estimated to have structural changes in the S protein (S:K854N) or the nonstructural open reading frame 9b (ORF9b:N36Y) with potential association with changes in protein function.

**Figure.**
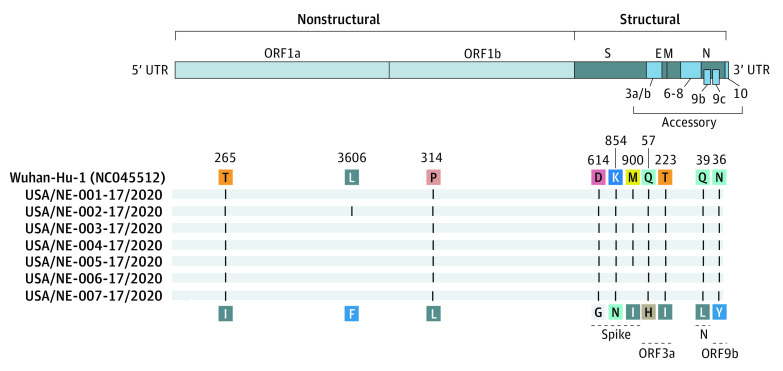
Sequence Alignment Between the 7 SARS-CoV-2 Strains and Wuhan Strain With Amino Acid Changes

## Discussion

Previous studies have demonstrated that sequence variations in the S protein may increase the infectivity and pathogenicity of SARS-CoV-2.^1,2^ The S:D614G variant has been associated with increased infectivity and transmissibility of SARS-CoV-2.^1^ Studies hypothesized that S:D614 within the first S protein subunit forms a key salt bridge with lysine at 854 in the second spike subunit when in the closed formation.^2^ Theoretically, S:D614G removes this important bridge resulting in a structural change estimated to enhance the ability of S protein to bind to angiotensin-converting enzyme 2 receptors (ACE2R).^2,5^ S:K854N has not been previously identified to our knowledge. In these 7 strains, a substitution for the positively charged lysine with asparagine, a polar AA, may restore the key salt bridge between S:D614G and S:K854N. This is estimated to result in a closed structural form and reduction of S protein binding ability to ACE2R, which may in turn result in a reduction in the virus ability to establish infection.

ORF9b antagonizes type I and type III interferons, which could reduce the cell’s ability to restrict viral replication.^6^ ORF9b variants may reduce pathogenicity.^6^ The ORF9b:N36Y variant has not been studied in vitro but it could introduce a structural change affecting the virus’ ability to antagonize interferons. Taken together, the reduced ability of the virus to propagate and to suppress interferon may explain why these individuals aged 75 years and older were asymptomatic and 5 of the 7 recovered without illness.

A limitation of this study is the small sample size, but surveillance for sequence variations is continuing to address this issue. In vitro studies are needed to determine whether the estimated outcomes of the variants described here associated with functional changes in cells with reductions in pathogenicity of the virus.
